# Preparation and Release Profiles *in Vitro/Vivo* of Galantamine Pamoate Loaded Poly (Lactideco-Glycolide) (PLGA) Microspheres

**DOI:** 10.3389/fphar.2020.619327

**Published:** 2021-03-08

**Authors:** Liping Du, Shankui Liu, Guizhou Hao, Li Zhang, Miaomiao Zhou, Yueqing Bao, Bing Ding, Qinyong Sun, Guimin Zhang

**Affiliations:** ^1^ Shandong Engineering Research Center of Complex Injectables, Shangdong New Time Pharmaceutical Co., Ltd., Linyi, China; ^2^ School of Biological Science and Technology, University of Jinan, Jinan, China; ^3^ International Pharmaceutical R&D Center, Lunan Pharmaceutical Group Co., Ltd., Linyi, China; ^4^ National Engineering Laboratory of High Level Expression in Mammalian Cells State Key Laboratory, Lunan Pharmaceutical Group Co., Ltd., Linyi, China

**Keywords:** galantamine, microspheres, physicochemical characterization, *in vitro* release, *in vivo* pharmacokinetic behavior

## Abstract

Patient’s poor compliance and the high risk of toxic effects limit the clinical use of galantamine hydrobromide. To overcome these drawbacks, the sustained-release galantamine pamoate microspheres (GLT-PM-MS) were successfully developed using an oil/water emulsion solvent evaporation method in this study. Physicochemical properties of GLT-PM-MS were carefully characterized, and the *in vitro* and *in vivo* drug release behaviors were well studied. Results showed that the morphology of optimized microspheres were spherical with smooth surfaces and core-shell interior structure. Mean particle size, drug loading and entrapment efficiency were 75.23 ± 1.79 μm, 28.01 ± 0.81% and 87.12 ± 2.71%, respectively. The developed GLT-PM-MS were found to have a sustained release for about 24 days *in vitro* and the plasma drug concentration remained stable for 17 days in rats. These results indicated that GLT-PM-MS could achieve the sustained drug release purpose and be used in clinical trial.

## Introduction

According to statistics of World Health Organization (WHO), there are currently 37 million people with dementia worldwide, and this number will be increased to 500 million by 2030. It is worth noting most of dementia patients are caused by Alzheimer’s disease (AD). Acetylcholinesterase inhibitors (AChEIs), such as donepezil, galantamine, and rivastigmine have been already on the market for the treatment of AD, which can improve or delay the progression of cognitive, behavioral, and functional deficits of patients. Among them, galantamine is one of the most popular anti-AD drugs. For one thing, it displayed potent acetylcholinesterase inhibition rate. For another, it could modulate the activity of nicotinic receptor and result in the associated conformational change (Samochocki et al., 2000; Farlow, 2001). Due to the poor stability of galantamine, the active pharmaceutical ingredient (API) of its tablets and capsules is galantamine hydrobromide (Zarotsky et al., 2003). However, there are still two major drawbacks for its conventional oral drug formulation. One is the patient’s poor therapeutic effect, owing to their bad memory and gastrointestinal side effects, such as nausea, vomiting and diarrhea. The other is the high risk of toxic effects resulted from the sharp increase of drug concentration in a short time (Dengiz and Kershaw, 2004; Evans et al., 2004). Therefore, development of a long-acting formulation of galantamine is urgently needed for the treatment of AD.

Among the current drug delivery systems, microspheres are particularly obvious by providing sustained-release of drugs over a long period of time ranging from a few days to months, decreasing dosing frequency, simplifying the drug regimen (Ford et al., 2013; Chaudhary et al., 2019). Up to now, 14 kinds of drugs have been developed the formulation of microspheres and used for clinical use. This drug delivery system has good biocompatibility, because its carrier materials are composed of biodegradable macromolecules such as polymers (cellulose, chitin, and chitosan, etc.) and naturally occurring monomers (lactic and glycolic acids) (Freiberg and Zhu, 2004). The most commonly used polymer is poly (lactideco-glycolide) (PLGA), which has been approved by US Food and Drug Administration (FDA). Note that its molecular weight and mole ratio of monomers can directly affect the drug release behaviors of microspheres (Janoria and Mitra, 2007; Su et al., 2011). The mainly prepared methods of microspheres are the emulsion solvent evaporation, spray drying and phase separation (Cleland, 1998; Giunchedi et al., 2000; Liu et al., 2010; Ramazani et al., 2016).

The purpose of this study is to develop the microspheres of galantamine, which can release the drug slowly over a period of 2–4 weeks after single dose, and reduce drug dosing with effective drug utilization. However, the poor solubility of galantamine hydrobromide (5.17 mg/ml, 25°C) limits the drug loading of its microspheres, which is usually tens or hundreds of times compared with its conventional formulation (Marco-Contelles et al., 2005; Saigal et al., 2013). Pamoic acid has been reported that it could be used as hydrophobic counterion to reduce the solubility of hydrophilic drugs (Ashton et al., 2016; Li et al., 2018). The increased organic phase solubility of the hydrophobic counterion can increase the drug loading of microspheres, which further leads to the reduced dosing frequency (Song et al., 2016). Therefore, galantamine pamoate (GLT-PM) was synthesized and loaded on the PLGA microspheres, which were prepared using an oil/water emulsion solvent evaporation method. To achieve the aimed release period of 2–4 weeks of microspheres, the molecular weight of PLGA and its mole ratio of monomers were 15 kDa and 75:25, respectively (Janoria and Mitra, 2007; Su et al., 2011). Plackett–Burman design (PBD) was applied for the optimization of formulation and process parameters. Finally, the optimized GLT-PM microspheres (GLT-PM-MS) were evaluated for their morphology, particle size, drug loading and entrapment efficiency, *in vitro* drug release, *in vivo* drug release in rats and stability.

## Materials and Methods

### Materials

Galantamine hydrobromide (purity ∼99.7%, Suzhou My Land Pharm and Nutrition Inc. Co., Ltd., Suzhou, China) of pharmaceutical grade was used without further purication. Galantamine hydrobromide reference standard (USP, purity ∼99.7%) was also purchased. Pamoic acid reference standard (purity ∼99.8%) was purchased from Chinese National Institutes for Food and Drug Control. PLGA (lactide:glycolide 75:25, Mw 15 kDa) was purchased from Shandong Institute of Medical Instruments (Jinan, China). Pamoic acid disodium salt monohydrate was purchased from TCI Shanghai Dev. Co, Ltd (Shanghai, China). Poly (vinyl alcohol) (PVA-1788) was obtained from Jiangxi Alpha Hi-Tech Pharmaceutical Co., Ltd (Pingxiang, China). Dichloromethane (DCM), Methanol and Benzyl Alcohol (BnOH) were supplied by Merck. Dimethyl sulfoxide-D_6_ (DMSO-D_6_) was purchased from Cambridge Isotopes Laboratories, Inc. All other reagents were analytical grade and obtained from Sinopharm Chemicals Reagent Company (Shanghai, China). Purified water was used throughout the experiment.

### Preparation of GLT-PM

Firstly, pamoic acid disodium salt monohydrate (5.0 g, 10.9 mmol) and galantamine hydrobromide (6.7 g, 18.1 mmol) were separately dissolved in 500 ml deionized water. Then they were mixed and stirred for 2 h at room temperature. During this process, the insoluble substance was precipitated out from the mixed solutions. Subsequently, the precipitation was filtrated and washed with deionized water three times (4–8°C, 200 ml each time). Finally, the light yellow color powder was obtained after heat treatment in vacuum (Fine Chem, 2015).

### Characterization of GLT-PM

#### Determination of Purity

The purity of GLT-PM was determined by HPLC method (Pharmacopoeia of the People’s Republic of China, 2015). Agilent 1260 HPLC (Agilent Technologies, Palo Alto, CA, United States) was used, which was equipped with a reverse phase column (4.6 * 250mm, 5μm, Zorbax Eclipse SB-C18) and a UV detector (Agilent Technologies, Palo Alto, CA, United States). The mobile phase was consisted of methanol/0.05 mol/L triethylamine buffer solution (adjust pH to 6.0 with 0.5 mol/L phosphoric acid) (25/75, *v/v*). The flow rate was 1.0 ml/min. The detection wavelength was 289 nm at column temperature of 40°C, and the injection volume was 20 μL. The calibration curve showed good linearity over a concentration range of 10–100 μg/ml. The limits of detection and quantification were 40ng/ml and 100ng/ml, respectively.

#### NMR

Experiment was performed in Varian AS 400 FT-NMR spectrometer (California, United States) operating at a frequency 400.13 MHz for ^1^H NMR, equipped with 5 mm ID probe and 5 mm ASW probe. 10 mg of sample powder was weighed and transferred into a NMR tube, with 0.55 ml of DMSO-D_6_ then added. Sample was sonicated at room temperature until completely dissolved.

#### Differential Scanning Calorimetry

The thermal properties of GLT-PM were analyzed by comparison with galantamine and pamoic acid, using a DSC-200F3 thermal analyzer (Netzsch L, Ltd., Hanao, Germany). Samples were separately placed in an aluminum pan and sealed with a porous cap. Then they were heated from 25°C to 400°C at a heating rate of 5°C/min under nitrogen protection.

#### Powder X-Ray Diffraction

Powder X-ray diffraction analysis of GLT-PM was characterized with comparison of galantamine and pamoic acid by an empyrean X-ray powder diffraction equipment (Panalytical Ltd., Almelo, Holland) using CuKα radiation, a counter speed 2 deg/minute, and a range of intensity measurement of ∼1,000.

#### Determination of Solubility of GLT-PM

∼200 mg of GLT-PM and galantamine hydrobromide were separately added into centrifuge tubes with 0.5 ml different solvents such as: deionized water, DCM, Methanol and BnOH. Then they were shaken for 24 h with 100 rpm at 25°C. At last, the supernatant was directly injected into HPLC after high speed centrifugation (3,000 rpm for 10 min).

### Preparation of GLT-PM-MS

An oil-in-water emulsion solvent evaporation method was used to prepare GLT-PM-MS (Cleland, 1998; Freiberg and Zhu, 2004; Chaudhary et al., 2019). Firstly, PLGA and GLT-PM were dissolved in 4 ml mixed solvents (DCM/BnOH, 4:1, *v/v*) in turn, which formed the organic phase. Secondly, PVA was dissolved in deionized water, which was used as the aqueous phase. Thirdly, the organic phase was subsequently dispersed into the aqueous phase, and L5M homogenizer was used to form a homogeneous emulsion during this process (Silverson Machines Ltd., England). Fourthly, the emulsion was stirred to evaporate DCM. Fifthly, the solidified microspheres were collected by filtration and added into the next solution (10% ethanol solution, *v/v*) to remove BnOH. Then, above microspheres were collected again and washed with 600 ml deionized water three times to remove the residual PVA. Finally, the collected microspheres were treated by freeze-drying and stored at 2–8°C.

The freeze-drying curve was as follows: the board temperature was maintained at −40°C for 3 h during the freezing step. And then the temperature was raised to −10°C and kept for 18 h with the vacuum degree 0.4 mbar during the primary drying step. In the end, the temperature was raised again to 20°C and kept for 24 h with zero vacuum degree during the secondary drying step.

### Experimental Design for the Preparation of Microspheres

As the number of factors affecting properties of GLT-PM-MS is more than 8, Plackett-Burman two-level partial factorial design (PBD) was used to evaluate the significant process parameters to reduce the times of experiments. As a result, an optimal solution of experimental parameters was obtained initially by response optimizer (Jiang et al., 2014). The independent variables were as follows: concentration of PLGA (X_1_), API (X_2_), PVA (X_3_) and mannitol (X_4_), volume ratio of aqueous phase to organic phase (X_5_), temperature of organic phase (X_6_) and aqueous phase (X_7_), emulsification speed (X_8_) and time (X_9_), temperature of solvent evaporation (X_10_), stirring speed (X_11_) and time (X_12_) of solvent evaporation, volume ratio of aqueous phase to extraction solvent (X_13_), stirring speed of extraction (X_14_), time of extraction (X_15_), temperature of extraction (X_16_). Meanwhile, the responses of microspheres were the particle size (Y_1_) and entrapment efficiency (Y_2_). Each independent variable was tested at two levels, a high and a low level, as shown in Table 1. The design matrix was generated by the Minitab Statistical 15 software (Minitab, LLC., Pennsylvania, United States) and consisted of 20 experimental runs. All the experiments were performed in triplicate at random in order to avoid the bias.

**TABLE 1:** The independent variables and their levels in Plackett–Burman design.

Independent variables	Levels	
	Low	High
X_1_-PLGA concentration (mg/ml)	150	700
X_2_-API concentration (mg/ml)	75	250
X_3_-PVA concentration (%, w/v)	0.1	3.0
X_4_-Mannitol concentration (%, w/v)	1	5.0
X_5_-Volume ratio of aqueous phase to organic phase	50	200
X_6_-Temperature of organic phase (°C)	10	25
X_7_-Temperature of aqueous phase (°C)	10	30
X_8_-Emulsification speed (rpm)	800	3,000
X_9_-Emulsification time (s)	10	60
X_10_-Temperature of solvent evaporation (°C)	25	45
X_11_-Stirring speed of solvent evaporation (rpm)	100	500
X_12_-Time of solvent evaporation (h)	2	5
X_13_-Volume ratio of aqueous phase to extraction solvent	1	2
X_14_-Stirring speed of extraction (rpm)	100	500
X_15_-Time of extraction (h)	0.5	3
X_16_-Temperature of extraction (°C)	10	30

The main effect of each independent variable was simply calculated as the difference between the average of measurement made at the high level and the average of measurements observed at low level of that factor. PBD is based on the first order model.

$$Z=b_{0}+\sum b_{i}x_{i},$$

Where *Z* is the response (particle size and entrapment efficiency), *b*_0_ is the model intercept and bi is the linear coefficient and Xi is the level of the independent variable (Kishore and Reddy, 2012).

### Preparation of the Diluents for Microspheres

This preparation was carried out in super-clean laboratory. Firstly, sodium carboxymethylcellulose (2.5 g) was dissolved at 80°C in 500 ml water for injection and stirred at 800 rpm for 2 h. Then this solution was cooled to room temperature and mixed with polysorbate 80 (0.5 g) and mannitol (25 g). After this, above solution was filtrated with 0.22 μm membrane and sub-packed in 7 ml vials.

### Characterization of GLT-PM-MS

#### Scanning Electron Microscope Analysis

The morphology and surface of optimized microspheres were characterized by a COXEM EM-30 Plus^+^ microscopy (Beijing Opton Optical Technology Co., Ltd., Beijing, China). The analyzed microspheres were coated with a thin layer of gold, using an ETD-2000 auto fine coater (Beijing Elaborate Technology Development Ltd., Beijing, China). The amperage and sputtering time of coating process were 10 mA and 80 s, respectively. Images were obtained at 10 kV of acceleration voltage by SEM. In addition, the cross-section of GLT-PM-MS was also observed, which was cut with a razor blade.

#### Particle Size Analysis

The particle size of optimized microspheres was determined by Malvern Mastersizer 3,000 (Malvern Panalytical Ltd., Malvern, England). Firstly, approximately 100 mg of samples were dispersed in 10 ml deionized water by ultrasonic dispersion. Then, the prepared suspension was added into the sample cup, and samples were detected at a revolution speed of 2,500 rpm. Note that the level of obscuration was between 10% and 20% throughout testing process. Besides this, the particle size distribution was calculated by the following equation.

$$\text{Span}=\frac{D_{90}-D_{10}}{D_{50}},$$ (1)

Where *D*_90_ is the cumulative particle size distribution at 90% of the volume, *D*_10_ is at 10%, and *D*_50_ is at 50%. For span values ≤ 5, the size distribution is considered to be narrow (Lin et al., 2018).

#### Drug Loading and Entrapment Efficiency

The content of API encapsulated in GLT-PM-MS was determined using the following method (Freiberg and Zhu, 2004; Zhang et al., 2019). Firstly, ∼20 mg of microspheres was dissolved in 1.0 ml DMSO. And then 0.1 mol/L hydrochloricacid solution https://fanyi.baidu.com/?aldtype=16047-zh/en/javascript:void(0); was added and mixed to the mark of 50 ml volumetric flask. After 10 min’ standing, the suspension was filtered through a 0.22 μm membrane filter, and the filtrate was analyzed to determine the concentration of drug by HPLC method.

The drug loading and entrapment efficiency were calculated using the following equations:

$$\text{Drug loading }\left(\%\right)=\frac{\text{Weight of drug in microspheres}}{\text{Weight of microspheres}}\times 100\%,$$ (2)

$$\text{Theoretical drug loading }\left(\%\right)=\frac{\text{Weight of API}}{\text{Weight of API }+\text{Weight of PLGA}}\times 100\text{\%,}$$ (3)

$$\text{Entrapment efficiency }\left(\%\right)=\frac{\text{Drug loading}}{\text{Theoretical drug loading}}\times 100\%.$$ (4)

In addition, DSC and powder X-ray diffraction of GLT-PM-MS were also characterized according to *Differential Scanning Calorimetry* and *Powder X-Ray Diffraction* methods, individually.

### *In vitro* Drug Release

The drug release study of optimized microspheres was carried out on a USP Apparatus 4 (Sotax CE7smart, and CY7 piston pump, Sotax, Horsham, United States) in closed mode (Zolnik et al., 2005). ∼60 mg of microspheres were dispersed in flow through cells (12-mm diameter, packed with 1 mm glass beads), and 250 ml of PBS buffer solution (pH 7.4, containing 0.1% SDS and 0.5% sodium azide, *w/v*) was circulated through a 0.45 mm fiberglass. The mechanism of drug release of PLGA microspheres has been reported to be temperature dependent, and it was a diffusion-controlled process at temperatures above Tg (Aso et al., 1994). Based on this, the release temperature of this study was selected at 45°C with a flow rate of 8 ml/min. Samples of 1.0 ml were withdraw and replaced with same volume fresh PBS medium at the following intervals (2 h and 1 day, 2 days, 3 days, 4 days, 6 days, 8 days, 10 days, 12 days, 14 days, 16 days, 18 days, 20 days, 22 days, 24 days). Samples were analyzed by HPLC method. All the measurements were conducted in triplicate.

### *In vivo* Drug Release

#### Animals and Drug Administration

Healthy Sprague-Dawley (SD) rats weighing 200–210 g were supplied by the Experimental Animal Breeding Center of Jinan Pengyue Co., Ltd. (Jinan, China). Before the drug administration, all rats were fasted for 12 h. But they were free access to diet and water throughout the study (Zhang et al., 2019). This study conformed to the Guide for the Care and Use of Laboratory Animals.

The study groups were carried out with twelve rats, which were randomly divided into two groups each with six rats. Group I received GLT-PM-MS suspension in diluent at a single dose of 10 mg/kg (calculated on galantamine) by intramuscular injection (*i.m.*). Group II also received GLT-PM-MS suspension in diluent at a single dose of 20 mg/kg (calculated on galantamine) by *i.m.*. The blood samples of both groups were obtained via the orbital venous plexus and collected into heparinized tubes before and after dosing at 1 h, 3 h, 6 h, 12 h, 1 day, 2 days, 3 days, 4 days, 6 days, 8 days, 10 days, 12 days, 114 days, 16 days, 18 days, 20 days. Other eighteen rats were randomly divided into the placebo group, negative group and oral administration group of six rats each. The placebo group received black microspheres suspension in diluent at a single dose of 20 mg/kg by *i.m.*, and the blood samples of this group were obtained the same as group I. The negative group was also carried out without any treatment, and the blood samples of this group were obtained the same as group I. The oral administration group was performed with GLT-PM (24 mg/kg), which was infused to stomach of rats. The blood samples of oral administration group were obtained before and after dosing at 15 min, 30 min, 45 min, 1 h, 1.5 h, 2 h, 3 h, 4 h, 6 h, 8 h, 12 h, 24 h, 48 h, 72 h. All blood samples were centrifuged immediately at 4,500 rpm for 10 min, and the plasma samples were separated into EP tubes and stored at −80°C until analysis (Jiang et al., 2014; Zhang et al., 2019).

#### Sample Preparation and Quantification

The plasma concentration of galantamine was determined by HPLC-ESI-MS/MS analysis. After thawing at room temperature, 50 μL plasma samples were separately added into a 1.5°ml centrifuge tube with the addition of 150 μL of acetonitrile. This mixture was vortex mixed for 1 min and centrifuged at 12700 rpm for 10 min. Then 100 μL supernate was mixed with 100 μL deionized water, and they was injected into vials for analysis (Zhang et al., 2019).

AB Sciex QTrap 6,500 + triple-quadrupole tandem mass spectrometer (Applied Biosystem Inc., Foster City, Calif, United States) was connected to Waters Acquity UPLC H-Class HPLC system (Waters Inc., Milford, Massachusetts, United States) via electro-spray ionization (ESI) interface. LC separation was performed on a Waters BEH C8 column (2.1 × 50 mm I.D., 1.7 μm) with mobile phase A (1 mmol/L ammonium acetate solution) and phase B (mixture of acetonitrile and methanol, 4:1, *v/v*) at 40°C, which was used with gradient elution as shown in Table 2 (Suresha et al., 2014). Quantitative analyses were performed in positive ion mode on a 6500 Q-trap mass analyzer (AB Sciex, Framingham, MA) coupled with electrospray ion source. Multiple reaction monitoring (MRM) mode was used for the quantification with ion spray voltage set at 5500 V, curtain gas 35 psi, ion source gas 150 psi, ion source gas 250 psi, and the interface heater temperature 450°C. Entrance potential (EP), collision cell exit potential (CXP), and declustering potential (DP) were set at 10, 11, and 91 V, respectively. The MRM transitions monitored in the experiments is m/z 288.2 ([M + H]) for galantamine (Suresha et al., 2014; de Paiva et al., 2019).

**TABLE 2:** The gradient elution of mobile phase of chromatography.

Total time (min)	Flow rate (µL/min)	Ammonium acetate (%)	Methanol (%)
0	600	95	5
0.5	600	95	5
1	600	20	80
1.5	600	20	80
1.51	600	95	5
3	600	95	5

#### Analysis of Pharmacokinetic Date

The plasma concentration-time data were analyzed with non-compartmental model by Phoenix WinNonlin 8.1 software (Pharsight Corporation, United States) to obtain the pharmacokinetic parameters, which included the area under the plasma concentration-time curve (AUC), half-life (T_1/2_) and mean residence time (MRT). The maximum plasma drug concentration (C_max_) and the time required to reach C_max_ (T_max_) were directly read from the plasma concentration-time data.

### Stability

Stress stability experiment of optimized microspheres was carried out to investigate the stability of GLT-PM-MS and provide a theory basis for its storage. GLT-PM-MS were firstly packed in 7 ml brown vials with 200 mg microspheres per vial. Then these vials were divided into four groups to investigate the stability of GLT-PM-MS at different conditions. Group I was placed into a 40 ± 2°C chamber (Binder Company, Germany), Group II was placed into a 25 ± 2°C chamber (Binder Company, Germany), Group III was placed into a 90 ± 5% RH chamber (Binder Company, Germany) and Group IV was placed into a light incubator (Binder Company, Germany) with 4,500 ± 500 lx. On the 10^th^ day, all samples were taken out for analysis of their appearance and drug content.

## Results and Discussion

### Characterization of GLT-PM

**FIGURE 1:**
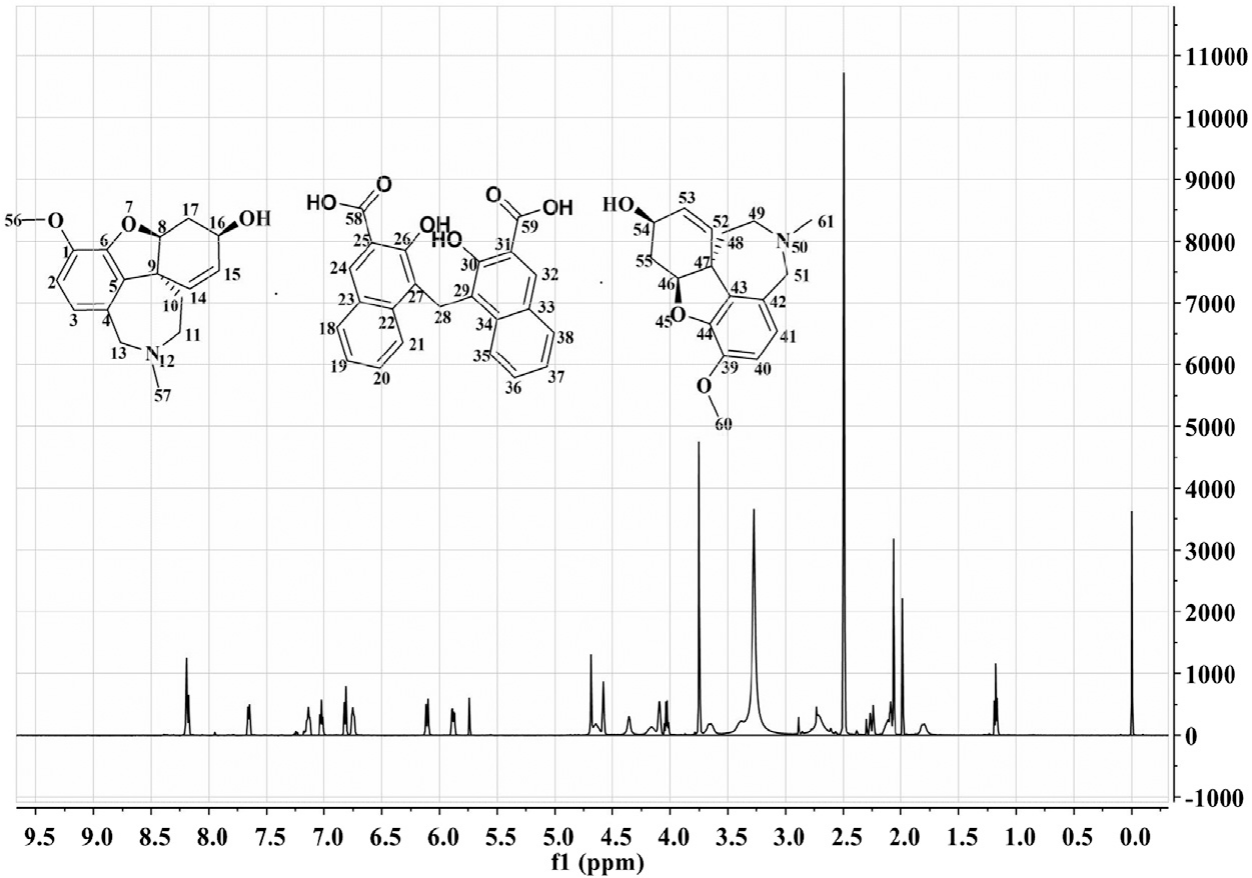
The possible structure of GLT-PM and its ^1^H-NMR spectra.

HPLC results showed that GLT-PM was freed into two compounds in mobile phase, which indicated the combination of galantamine and pamoic acid were intermolecular interactions. Retention times of galantamine and pamoic acid were at 5 min and 16 min, respectively. They were analyzed with their reference standards both quantitatively and qualitatively. The contents of galantamine and pamoic acid were 59.5% and 40.3%, individually. According to the molar mass of above two compounds, the mole ratio of galantamine and pamoic acid was 2:1 in GLT-PM. Based on this, the inferred structure of GLT-PM was shown in Figure 1.

1H-NMR spectra was used to characterize the structure of GLT-PM, which was also displayed in Figure 1. The shifts of protons were confirmed as following: δ_24_,_32_ 8.19 ppm (s, 2H), δ_21_,_35_ 8.14 ppm (d, 2H), δ_18_,_38_ 7.65 ppm (d, 2H), δ_19_,_37_ 7.13 ppm (m, 2H), δ_20_,_36_ 7.02 ppm (m, 2H), δ_2_,_40_ 6.82 ppm (d, 2H), δ_3_,_41_ 6.75 ppm (d, 2H), δ_15_,_53_ 6.10 ppm (m, 2H), δ_14_,_52_ 5.88 ppm (s, 2H), δ_13a_,_51a,28_ 4.68 ppm (d, 4H), δ_13b_,_51b_ 4.58 ppm (d, 2H), δ_16_,_54_ 4.02 ppm (t, 2H), δ_56_,_60_ 3.75 ppm (s, 6H), δ_11a_,_49a_ 2.71 ppm (m, 2H), δ_11b_,_49b_ 2.60 ppm (m, 2H), δ_17a_,_55a_ 2.30 ppm (d, 2H), δ_57_,_61_ 2.26 ppm (s, 6H), δ_17b_,_55b_ 2.10 ppm (d, 2H), δ_10a_,_48a_ 1.96 ppm (m, 2H), δ_10b_,_48b_ 1.17 ppm (m, 2H). This result confirmed the formation of the complex of GLT-PM.

**FIGURE 2:**
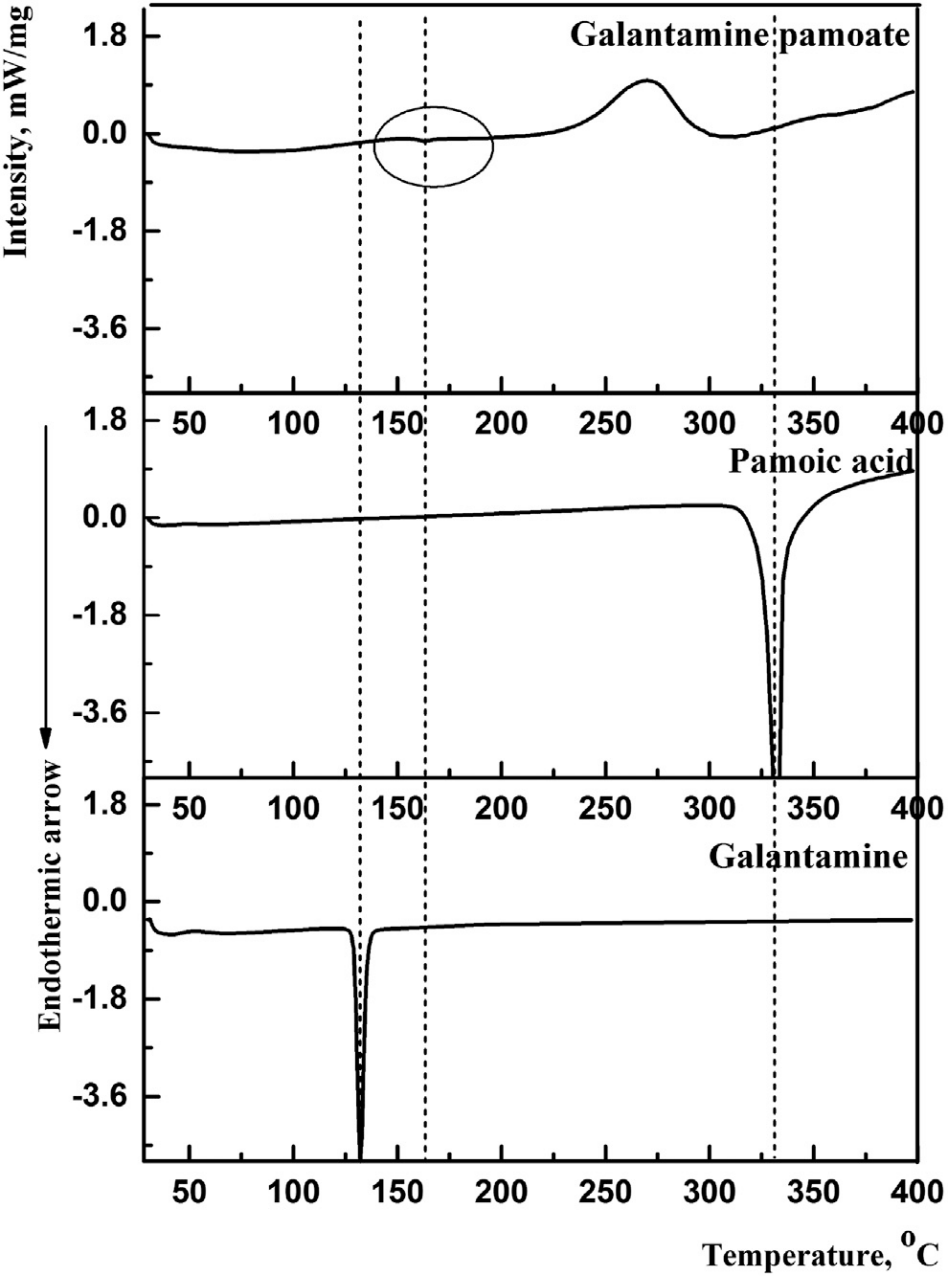
DSC curves of galantamine, pamoic acid and GLT-PM.

By compared with galantamine hydrobromide and pamoic acid, the thermal traces and behaviors of GLT-PM were investigated. DSC curves were presented in Figure 2. The melting peaks of galantamine hydrobromide and pamoic acid were separately at 132.3°C and 330.8°C. For galantamin pamoate, it was only observed a very weak endothermic peak at 162.2°C and a broad exothermic peak at 270.0°C. The disappearance of endothermic peaks of galantamine hydrobromide and pamoic acid indicated a different complex had been formed. The small area of endothermic peak suggested the form of GLT-PM was amorphous, which was resulted from the melting of this compound.

**FIGURE 3:**
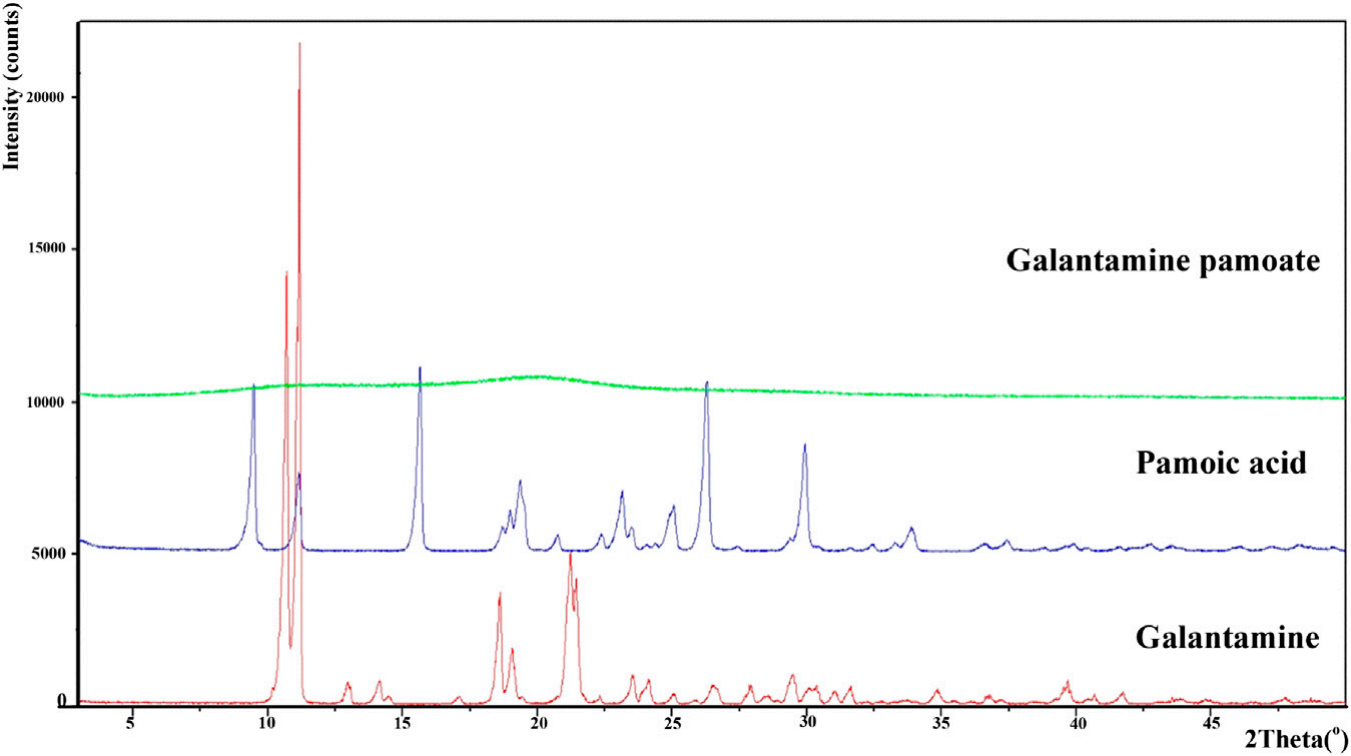
X-ray diffractogram of galantamine, pamoic acid and GLT-PM.

Powder X-ray diffraction of galantamine hydrobromide, pamoic acid and GLT-PM were displayed in Figure 3. The main characteristic peaks of galantamine and pamoic acid were observed in the X-ray patterns, indicating the crystal structure of these two compounds. However, X-ray diffraction pattern of GLT-PM was blunt without any characteristic peak, indicating an amorphous form of this complex.

In addition, the solubility of GLT-PM was investigated, and results were shown in Table 3. By compared with galantamine hydrobromide, the solubility of GLT-PM was significantly reduced in deionized water. Meanwhile, a higher solubility of GLT-PM was observed in BnOH, indicating a higher lipophilicity of this complex.

**TABLE 3:** The solubility of GLT-PM and galantamine hydrobromide in different solvents (25°C, mean ± SD, *n* = 3).

Compound	Deionized water (mg/ml)	Dichloromethane (mg/ml)	Methanol (mg/ml)	Benzyl alcohol (mg/ml)
GLT-PM	0.20 ± 0.02	8.72 ± 0.52	1.01 ± 0.21	357.30 ± 0.21
Galantamine hydrobromide	4.32 ± 0.37	0.32 ± 0.03	0.82 ± 0.09	1.57 ± 0.19

### Analysis of Experimental Design Data

The particle size and entrapment efficiency of microspheres have important effects on the drug release behaviors. In addition, the particle size can affect the syringeability of microspheres, and entrapment efficiency can decide the dosage of products. Therefore, the particle size and entrapment efficiency were separately used as responses of *Y*_1_ and *Y*_2_ to evaluate the main factors of sixteen independent variables. PBD was applied on the design of experiments for 16 factors at two levels, and a total of 20 experiments were performed and their responses were summarized in Table 4. Main effects to all the observed responses were analyzed using Minitab 15 software among the tested factors. Results showed the following factors are significant to the mean particle size (*Y*_1_), such as: PLGA concentration (X_1_), PVA concentration (X_3_), mannitol concentration (X_4_), stirring speed of solvent evaporation (X_11_). For entrapment efficiency (*Y*_2_), its significant factors include PLGA concentration (X_1_), PVA concentration (X_3_), mannitol concentration (X_4_), volume ratio of aqueous phase to organic phase (X_5_), temperature of organic phase (X_6_), temperature of aqueous phase (X_7_), emulsification speed (X_8_), emulsification time (X_9_), temperature of solvent evaporation (X_10_), stirring speed of solvent evaporation (X_11_), time of solvent evaporation (X_12_), volume ratio of aqueous phase and extraction solvent (X_13_) and time of extraction (X_15_). No abnormalities were found among them. Regression analysis for responses *Y*_1_ and *Y*_2_ was summarized in Table 5. It was found the values of *p* and *R* ^*2*^ suggest the appropriate controllable variables were selected. The significant factors can well explain the variation of responses, where *R* ^*2*^ is >80% (Tang et al., 2007).

**TABLE 4:** Plackett–Burman experimental design and the observed responses.

Run	Independent variables																Dependent variables	
	X_1_ (mg/ml)	X_2_ (mg/ml)	X_3_ (%)	X_4_	X_5_	X_6_ (°C)	X_7_ (°C)	X_8_ (rpm)	X_9_ (s)	X_10_ (°C)	X_11_ (rpm)	X_12_ (h)	X_13_	X_14_ (rpm)	X_15_ (h)	X_16_ (°C)	*Y*_1_ (μm)	*Y*_2_ (%)
1	700	250	3.0	1	200	10	30	800	60	25	100	2	1	500	0.5	30	128.0 ± 3.0	98.23 ± 0.31
2	700	250	0.1	1	50	10	30	3,000	10	45	100	5	2	500	0.5	10	94.4 ± 2.5	95.05 ± 0.43
3	700	75	0.1	1	200	10	10	800	60	45	500	5	2	100	0.5	30	122.0 ± 1.9	98.95 ± 0.45
4	700	75	0.1	5	50	25	30	800	10	25	100	5	2	500	3.0	30	165.0 ± 2.8	56.16 ± 0.38
5	150	75	3.0	5	50	10	30	3,000	60	25	500	2	2	500	0.5	30	26.2 ± 2.0	21.22 ± 0.27
6	150	250	0.1	5	50	10	10	800	60	45	500	2	2	500	3.0	10	55.5 ± 3.1	45.04 ± 0.27
7	150	250	3.0	5	200	10	10	800	10	25	100	5	2	100	3.0	30	17.6 ± 1.8	30.92 ± 0.30
8	700	250	0.1	5	200	10	30	3,000	10	25	500	2	1	100	3.0	10	45.3 ± 2.2	87.24 ± 0.25
9	150	75	0.1	5	200	25	10	3,000	10	45	100	2	1	500	0.5	30	48.5 ± 1.7	59.27 ± 0.36
10	700	250	3.0	1	50	25	10	800	10	45	500	2	1	500	3.0	30	126.0 ± 2.9	97.09 ± 0.28
11	700	75	3.0	1	50	25	10	3,000	60	25	100	2	2	100	3.0	10	33.5 ± 2.1	68.15 ± 0.31
12	150	250	0.1	1	200	25	30	3,000	60	45	500	2	2	100	3.0	30	26.7 ± 3.2	47.91 ± 0.28
13	700	75	3.0	5	200	25	30	800	10	45	100	2	2	100	0.5	10	56.3 ± 2.2	66.81 ± 0.27
14	150	250	3.0	5	50	25	30	800	60	45	500	5	1	100	0.5	10	15.9 ± 1.8	28.66 ± 0.30
15	150	75	0.1	1	200	25	30	800	60	25	100	5	1	500	3.0	10	101.0 ± 3.4	63.36 ± 0.24
16	150	75	0.1	1	50	10	10	800	10	25	100	2	1	100	0.5	10	112.0 ± 2.9	70.67 ± 0.29
17	150	250	0.1	5	200	25	10	3,000	10	25	500	5	2	500	0.5	10	15.3 ± 2.0	62.33 ± 0.32
18	150	75	0.1	1	50	10	30	3,000	10	45	500	5	1	100	3.0	30	18.1 ± 2.9	46.15 ± 0.28
19	700	250	3.0	5	50	10	10	3,000	60	25	500	5	1	100	0.5	30	39.4 ± 2.2	67.51 ± 0.25
20	700	75	3.0	1	200	25	10	3,000	60	45	100	5	1	500	3.0	10	43.2 ± 2.1	69.59 ± 0.19

**TABLE 5:** Summary of results of regression analysis for responses.

Response	*R* ^*2*^	*R* ^*2*^ (predicted)	*R* ^*2*^ (adjusted)	p (main factors)
Mean particle size (*Y*_*1*_)	0.8761	0.7472	0.8319	0.044
Entrapment efficiency (*Y*_*2*_)	0.9963	0.9767	0.8098	0.004

Furthermore, an optimization of the independent variables by the desirability function was obtained using response optimizer of Minitab Statistical 15 software. The desirable particle size of optimum formulation of microspheres was 70 μm, which had good syringeability and couldn’t lead to quickly drug release. For the entrapment efficiency, the higher it is, the lower the dosage of microspheres needs in clinic. So the maximum entrapment efficiency was selected as the desirability of optimum formulation. Then, the optimized formulation of independent variables was finally obtained, which was as follows: PLGA concentration (400 mg/ml), API concentration (190 mg/ml), mannitol concentration (4.2%, w/v), temperature of organic phase (10°C), PVA concentration (1.0%, w/v), volume ratio of aqueous phase to organic phase (150/1), emulsification speed (1,500 rpm), emulsification time (20 s), temperature of aqueous phase (20°C), stirring speed of solvent evaporation (150 rpm), time of solvent evaporation (2 h), temperature of solvent evaporation (40°C), volume ratio of aqueous phase and extraction solvent (1/1), stirring speed of extraction (150 rpm), time of extraction (0.5 h), temperature of extraction (20°C). These values predicted the particle size and entrapment efficiency were 80 μm and 98%, respectively. The composite desirability was 0.9973.

Above optimum levels of independent variables were carried out to confirm the validity of the proposed model, which were generated by the software. It was found the observed values of *Y*_1_ and *Y*_2_ were respectively 75.23 ± 1.79 μm and 87.12 ± 2.71%, which were discussed in Section *Analysis on DSC Thermogram* and Section *Analysis on Drug Loading and Entrapment Efficiency*. And their predict error showed low value (below 15%), which were 5.9% and 11.1% for *Y*_1_ and *Y*_2_. This result suggested the values of proposed model were in accordance with the predicted values. Therefore, the optimized formulation was used to prepare the microspheres of GLT-PM for the subsequent studies.

### Characterization of GLT-PM-MS

#### Analysis on Morphology

**FIGURE 4:**
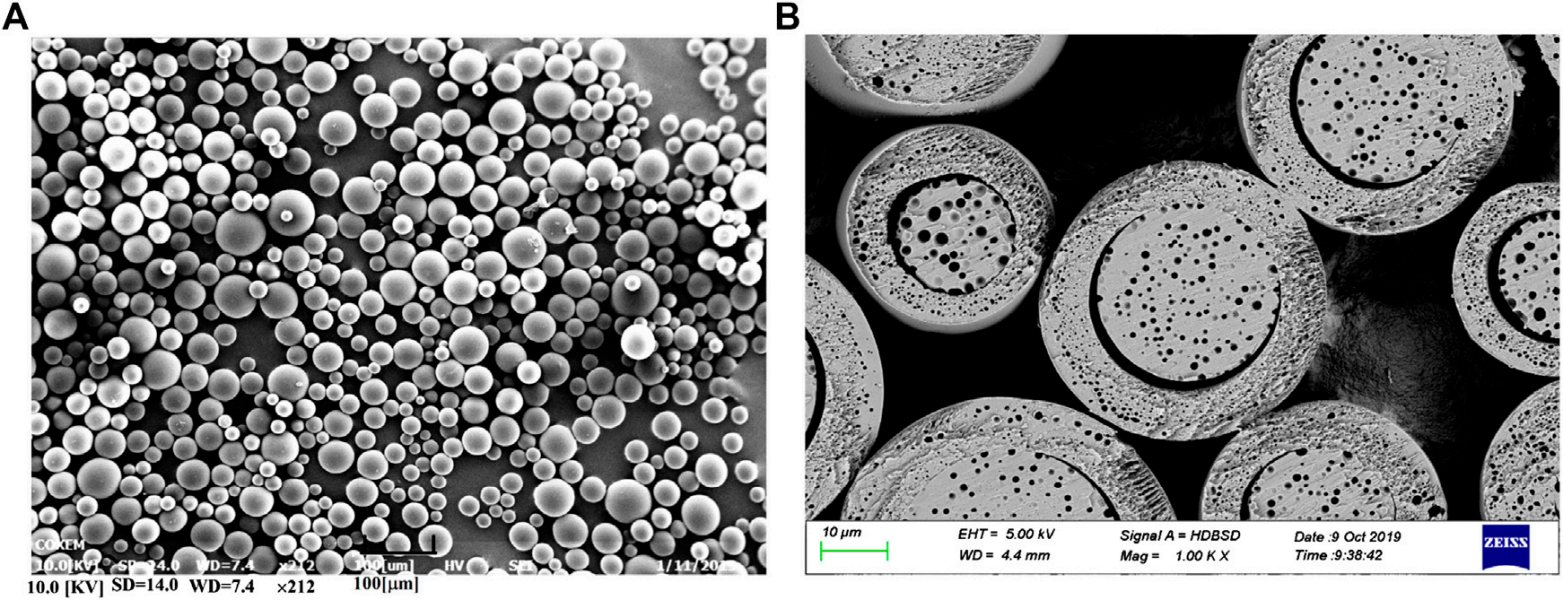
SEM images of GLT-PM-MS. **(A)**: the surface morphology, **(B)**: the cross-section morphology.

The morphological characteristic of optimized microspheres is illustrated in Figure 4. It was observed as-prepared microspheres showed a spherical shape with a smooth surface. Meanwhile, the cross-section exhibited a typical core-shell internal structure. This distinctive structure was referred to be related to its binary solvents system, which dissolved API and PLGA and formed the organic phase. During the preparation, the organic phase was sheared into small particles in aqueous phase and formed an emulsification. As the water-solubility of DCM and BnOH is different, this feature led to a different migrating rate of solvents from the inner of particles to the outer aqueous phase (Sui et al., 2012). Along with the removal of DCM from the surface of particles, the microspheres were quickly solidified and formed a shell. Differently, the interior of microspheres were slowly solidified along with the slowly removal of DCM and BnOH, which formed the core independent of the shell.

#### Analysis on Particle Size

According to report, the particle size has significant effects on the degradation of microspheres and *in vitro/in vivo* drug release behavior (Lin et al., 2018). Therefore, the particle size of optimized microspheres and its distribution had been investigated. It was found the mean particle size of microspheres was 75.23 ± 1.79 μm, and its span value was 1.8, indicating a narrow particle size distribution.

#### Analysis on DSC Thermogram

**FIGURE 5:**
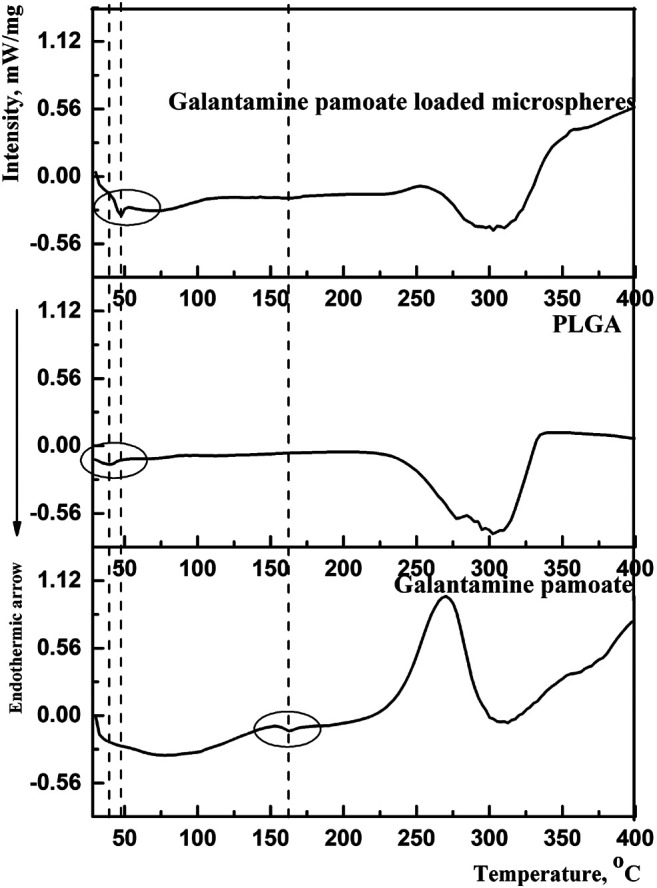
DSC curves of GLT-PM, PLGA and GLT-PM-MS.

DSC was carried out to study the molecular interactions of optimized microspheres between PLGA and GLT-PM. The thermal traces and behaviors of free GLT-PM, PLGA and as-prepared microspheres are represented in Figure 5. The weak endothermic peak of initial GLT-PM was at 162.2°C and degraded at 275.0°C. The glass temperature (Tg) of PLGA was at 39.5°C and degraded at 304.2°C. For optimized microspheres, it was observed the disappearance of endothermic and exothermic peaks of GLT-PM, indicating API had been uniformly dispersed in the microspheres (Bohr et al., 2011; Wang et al., 2015; Wang et al., 2016).

In addition, it was found that the Tg of PLGA shifted to a higher temperature (47.4°C) in drug-loaded microspheres. This variety was caused by the following two hands, one is the mobility of PLGA was broken by the existence of drug, the other is the molecular interactions, especially the hydrogen bonding, which had been formed between the hydrogen bond acceptors of GLT-PM and carboxyl terminal of PLGA (Guo et al., 2015). Based on above results, it was concluded GLT-PM had been well dispersed in all the prepared microspheres with the existence of hydrogen bond interaction.

#### Analysis on Powder X-Ray Diffraction

**FIGURE 6:**
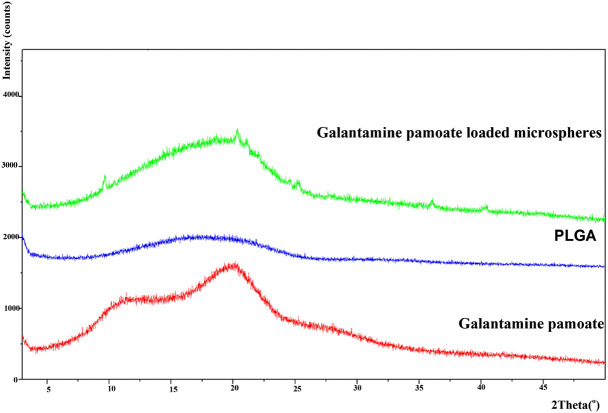
X-ray diffractogram of GLT-PM, PLGA and GLT-PM-MS.

The powder X-ray pattern of GLT-PM in formation of free molecule, PLGA and optimized microspheres are displayed in Figure 6. X-ray diffraction pattern of GLT-PM and API ware blunt without any characteristic peaks, indicating their formation being amorphous. And no characteristic diffraction peaks were found in the microspheres, indicating API had been dispersed in an amorphous formation without any crystal transformation during the preparation.

#### Analysis on Drug Loading and Entrapment Efficiency

Results of drug loading of optimized microspheres were determined to be 28.01 ± 0.81%. And high entrapment efficiency was observed, which was 87.12 ± 2.71% indicating the drug had been well encapsulated in microspheres. This result might be related to its special core-shell structure, which prevented the loss of drug from its inner to outer aqueous phase. In addition, BnOH has a higher boiling point and viscosity than DCM, and this led to more slowly diffusion of solvents and reducing the loss of drug from microspheres into the aqueous phase.

### *In vitro* Drug Release

**FIGURE 7:**
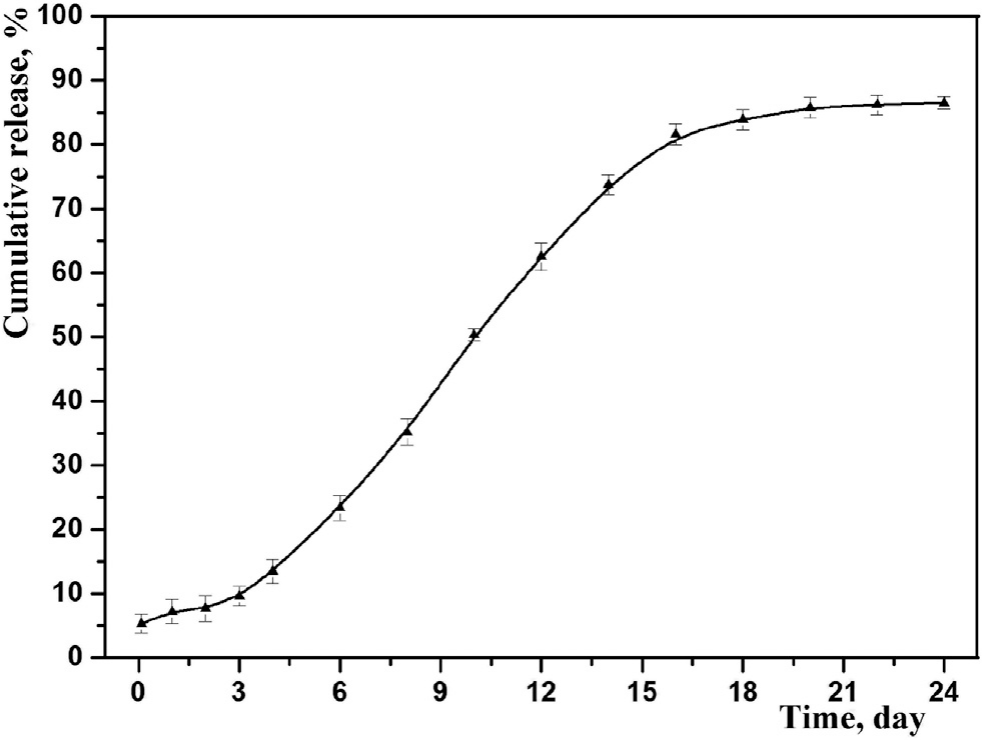
*In vitro* drug release profiles of GLT-PM-MS (mean ± SD, *n* = 3).

The drug cumulative release behavior of optimized microspheres is shown in Figure 7. As we can see, the prepared microspheres exhibited an obvious sustained release, and the accumulated amount of drug release was up to 87% in 3 weeks. At the same time, no obvious burst release was observed by studying the initial release thoroughly, indicating no exceed drug desorption on the outer surface of microspheres. This result is consistent with its core-shell structure, where the drug may mainly distribute among its core (Freiberg and Zhu, 2004).

To better understand, the mechanism of drug release from the PLGA microspheres was investigated by fitting above release data into zero order, first order, Higuchi and Korsmeyer-Peppas models. The correlation coefficient (*R* ^*2*^) obtained after linear regression on various kinetic models is listed in Table 6. It was found that *in vitro* release data was followed and supported by the first order model, because this model presented the highest value of *R* ^*2*^ (0.9609) with a release rate constant of k (0.0451). And the first-order release kinetic equation is as follows:

$$\;P=P_{0}\left(1-e^{-kt}\right),$$ (5)

Where *P* is the drug permeability coefficient during the polymer degradation, *P*_0_ is the drug permeability coefficient before the polymer began to degrade, and *k* was the degradation rate constant (Heller and Baker, 1980). It is not difficult to find the constant of degradation rate is independent of the initial permeability coefficient. In this study, the drug release rate was directly affected by themselves diffusion behaviors, and degradation rate of polymer was far smaller than the drug diffusion rate. Therefore, it is concluded that the mechanism of optimized microspheres was mainly controlled by the diffusion of drug through PLGA.

**TABLE 6:** The kinetic models simulated for the release behavior of GLT-PM-MS.

Model	Equation	*k*	*R* ^2^
Zero-order model	*y* = 4.118*x* + 3.545	*—*	0.9396
First-order kinetics model	*y* = −149.034 * exp (−*x*/22.157) + 145.144	0.0451	0.9609
Higuchi model	*y* = 22.853 * *x* ^1/2^—20.118	*—*	0.9222
Korsmeyer-Peppas model	*y* = 6.781 * *x* ^0.844^	*—*	0.9485

### *In vivo* Pharmacokinetic Study

**FIGURE 8:**
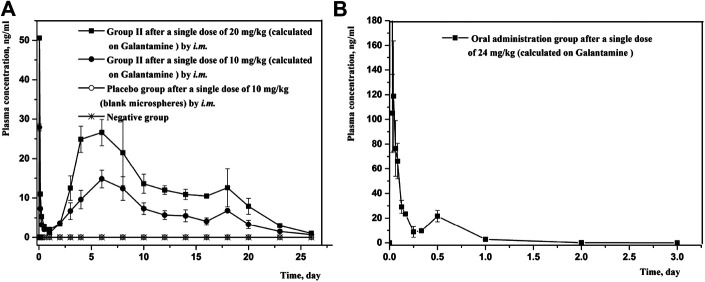
Mean plasma concentration-time profiles of rats following intramuscular injection and oral administration of GLT-PM-MS. (mean ± SD, *n* = 6).

Since the combination of galantamine and pamoic acid was intermolecular interactions in the synthesized complex, it was freed into two compounds after *i.m* or oral administration. Therefore, the plasma concentration of galantamine instead of GLT-PM was monitored to evaluate its *in vivo* pharmacokinetic behaviors. The mean plasma concentration-time profiles of galantamine were shown in Figure 8, which were obtained from rats after *i.m.* or oral administration of optimized microspheres. The main pharmacokinetic parameters calculated for galantamine were represented in Table 7. By compared with oral administration, the drug of GLT-PM-MS could be released slowly for about 3 weeks without sharp increase/decrease of drug concentration in a short time. And the plasma concentration of galantamine presented a dose-dependent manner. The activity of galantamine was reported to be maintained, when its plasma drug concentration is between 10 ng/ml and 118 ng/ml. And its minimal toxic concentration is 125 ng/ml (FDA. Pharmacology Review (s), 2001). Therefore, it is considered to be an optimal formulation, which maintains a plasma drug concentration between 10 ng/ml and 118 ng/ml *in vivo*. Based on this, stable plasma drug levels were observed from the 3rd day to the 19th day after a single dose of 0.4 ml (10 mg/ml), and the effective period of drug releasing was 17 days.

**TABLE 7:** Pharmacokinetic parameters of rats following *i.m.* administration of GLT-PM-MS (mean ± SD, *n* = 6).

Subject	T_1/2_ (d)	T_max_ (d)	C_max_ (ng/ml)	AUC_0-t_ (d * ng/mL)	AUC_0-∞_ (d * ng/mL)	MRT_0-t_ (d)
Group I	9.38 ± 2.25	7.00 ± 1.41	14.83 ± 2.23	217.10 ± 55.70	250.86 ± 41.40	8.82 ± 2.11
Group II	8.41 ± 1.73	7.00 ± 1.02	26.63 ± 3.31	323.91 ± 34.70	435.14 ± 59.93	9.39 ± 0.42
Placebo group	—	—	—	—	—	—
Negative group	—	—	—	—	—	—
Oral administration	0.18 ± 0.02	0.04 ± 0.01	119.02 ± 44.63	532.54 ± 73.08	532.91 ± 72.92	7.77 ± 0.95

### Stability

This experiment investigated the stability of GLT-PM-MA under high temperature, high humidity and strong light. The appearance of microspheres and drug content were used as the evaluation index. It was found the microspheres presented slight caking with the drug content decreasing from 28.01 ± 0.81% to 27.69 ± 0.52%, which was placed under 40 ± 2°C. This phenomenon might be caused by the storing temperature, which is close to the glass transition temperature of PLGA (Tg, 40–50°C) and led partial microspheres to melt. For the lower storing temperature (25°C), the appearance of microsphere was still slight yellow powder after storing 10 days, and the content of API was decreased to 27.60 ± 0.66%. For the conditions of both 90 ± 5% RH and 4,500 ± 500 lx, the appearance of microspheres was the same as their initial products, and their drug contents were respectively decreased to 27.09 ± 0.39% and 27.50 ± 0.09%. By the statistical analysis, the drug contents of GLT-PM-MS were no significant difference between stressed groups and initial products (*p* > 005). Based on above analysis, it is concluded that GLT-PM-MA has good stability, but it is temperature sensitive and need to be stored at low temperature (2–8°C).

## Conclusion

In this study, galantamine pamotate loaded microspheres have been successfully prepared using an oil-in-water emulsion solvent evaporation method. Plackett-Burman two-level partial factorial design was employed to optimize its formulation and process parameters. The optimized microspheres showed a spherical morphology with smooth surfaces and core-shell structure, and they also exhibited excellent drug loading, entrapment efficiency, sustained release behaviors in rats and good stability. In conclusion, the prepared microspheres of GLT-PM-MS are promising to prolong the extension time of galantamine *in vivo.* And it is expected to provide an optimized alternative for the treatment of AD.

## Data Availability

The raw data supporting the conclusions of this article will be made available by the authors, without undue reservation.
